# Photoinduced Dynamic Ligation in Metal–Organic Frameworks

**DOI:** 10.1021/jacs.3c12217

**Published:** 2023-12-27

**Authors:** Qingyu Ye, Daniel R. Cairnie, Diego Troya, Naveen Kumar, Xiaozhou Yang, Amanda J. Morris

**Affiliations:** † Department of Chemistry, Virginia Tech, Blacksburg, Virginia 24060, United States

## Abstract

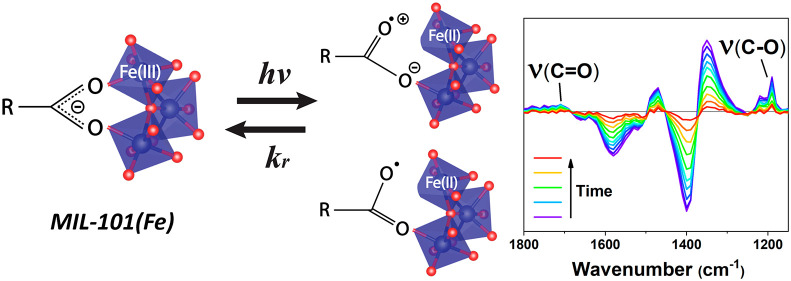

Metal organic frameworks (MOFs), a class of porous crystalline materials consisting of metal-based nodes and organic linkers, have emerged as a promising platform for photocatalysis due to their ultrahigh functional surface area, customizable topologies, and tunable energetics. While interesting photochemistry has been reported, the related photoinduced structural dynamics of MOFs remains unclear. The consensus is that the coordination bonds between MOF nodes and linkers are considered static during photoexcitation, while the open-metal sites on the nodes are taken as the key active sites for catalysis. In this work, through a complementary time-resolved visible and infrared (IR) spectroscopic investigation, along with computational studies, we report for the first time light-induced structural bond dissociation (COO-M) and reformation in an iron-oxo framework, MIL-101(Fe). The probed excited state displayed ligand-to-metal charge transfer (LMCT) characteristics and exhibited a *ca*. 30 μs lifetime. The incredibly long excited-state lifetime led us to probe potential structural rearrangements that facilitated charge separation in MIL-101(Fe). By probing the vibrational fingerprints of the carboxylate linker upon LMCT photoexcitation, we observed the reversible transition of the carboxylate-Fe bond from a bidentate bridging mode to a monodentate mode, indicating the partial dissociation of the carboxylate ligand. Importantly, the bidentate configuration is recovered on the same time scale of the excited state lifetimes as probed via visible transient absorption spectroscopy. The elucidated photoinduced configurational dynamics provides a foundation for an in-depth understanding of MOF-based photocatalytic mechanisms.

One of the limiting factors in molecular photocatalytic approaches for the transformation of highly stable compounds such as CO_2_ and H_2_O to useful commodities is the necessity to access highly reactive catalytic intermediates that are prone to degradation. In most common cases, catalyst degradation occurs via photoinduced ligand dissociation and dimerization, e.g., ruthenium polypyridyl catalysts.^1−6^ To mitigate the degree of dimerization, site isolation has been employed in molecular scaffolds, such as metal organic frameworks (MOFs), through known-molecular-catalyst incorporation or encapsulation to successfully prolong catalysis.^7−11^ However, even in these systems eventual photoinduced ligand dissociation and/or catalyst leaching can persist and limit desired long-term sustained catalysis.^7^ As an alternative to incorporation and encapsulation as MOF linkers or guests, recent results demonstrate that MOFs which feature open-metal sites on catalytic nodes can be active for photoinduced transformations.^12,13^ In these constructs, the presence of open-metal sites is usually considered independent of photoexcitation, as the frameworks are seen as configurationally static during the photocatalytic process (e.g., the metal-carboxylate coordination bonds in MOFs maintain their original bonding configuration at the ground state). However, recent work by Brozek et al. has suggested the dynamic nature of carboxylate-metal bonds in MOF ground states.^14^ Moreover, increasing instances support the notion of dynamic bonding in MOFs as justification for observed behaviors in MOF glasses.^15−21^ Consequently, a closer investigation is needed to provide us with more mechanistic details of the illuminated MOFs.

Fe-based MOFs have been investigated as potential candidates for important photocatalytic processes (e.g., CO_2_ reduction, H_2_O oxidation, H^+^ reduction), with ligand-to-metal charge transfer (LMCT) identified as the crucial transition that promotes reactivity.^22,23^ Lockard et al. confirmed the presence of an active LMCT state in the Fe-based MOF, MIL-100(Fe), via X-ray transient absorption (XTA) spectroscopy as a formal shift of electron density from the linker of the MOF to Fe^3+^ sites in the node, which exhibited a 3.6 μs lifetime.^23^ Notably, this long-lived charge transfer excited state is longer than most Fe-based photocatalytic systems.^23−26^ While the significant increase in Fe-oxo excited state lifetime displayed great promise for photocatalysis with Fe-based MOFs, the more detailed attributions for the charge migration and the longevity of the excited state remain unknown. In this communication, through a comprehensive study with visible transient absorption (VisTA) spectroscopy, time-resolved infrared (TRIR) spectroscopy, and computational simulations on a related Fe-based MOF, MIL-101(Fe) (composed of Fe_3_(μ_3_-O)Cl(H_2_O)_2_ nodes and benzene-1,4-dicarboxylate linkers; Figure 1), we correlate the LMCT transition to a photodissociation event (partial deligation of carboxylate linker from the Fe-oxo node) that promotes the long-lived LMCT state. The photoinduced ligand dissociation phenomenon reported here should promote a more detailed understanding of MOF photocatalysis and facilitate the rational design of future MOF-based photocatalytic systems.

**Figure 1:**
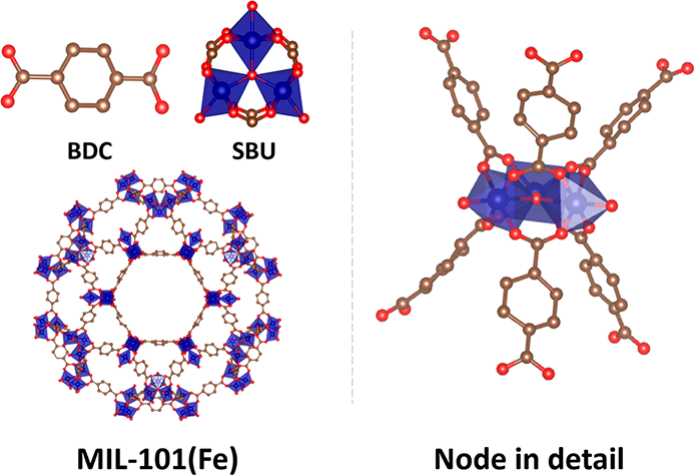
Schematic representation of MIL-101(Fe). The framework is composed of BDC linkers and secondary building units (SBU) containing an Fe-μ_3_-oxo cluster. The coordination between the linker and cluster exhibits a typical bidentate configuration.

MIL-101(Fe) was synthesized according to a published procedure (see Supporting Information).^27^ Bulk phase purity and the crystalline structure of MIL-101(Fe) were confirmed with powder X-ray diffraction (PXRD), which showed characteristic diffraction peaks in accordance with the simulated pattern (Figure S1). Microcrystalline particles of octahedral morphology can be clearly seen in the scanning electron microscopy (SEM) image (Figure S2).^28,29^ The thermogravimetric analysis (TGA) and N_2_ adsorption isotherm (Figures S3, S4) of the prepared MOF also agree well with the literature.^28^ Moreover, X-ray photoelectron spectroscopy (XPS) confirmed the presence of trivalent Fe and oxygen bridging the Fe that compose the Fe_3_(μ_3_-O)Cl(H_2_O)_2_ nodes (Fe_3_O) in MIL-101(Fe) (Figures S5–S7).^30^

Shown in Figure 2 (left) are the UV–vis absorption spectra of MIL-101(Fe) and its constituent linker, benzene-1,4-dicarboxylic acid (H_2_bdc), obtained via diffuse reflectance (DR) spectroscopy. At wavelengths below 300 nm, the electronic transitions in MIL-101(Fe) are primarily ligand-based, with π–π* and n−π* transitions occurring at 250 nm and *ca*. 280 nm, respectively. In the visible region, from *ca*. 450 nm onward, MIL-101(Fe) exhibits weak ligand-field (*d–d*) transitions occurring at the Fe metal center.^26,31−33^ The region of interest, 350–450 nm, shows a strong transition assigned to an LMCT band.^23^

**Figure 2:**
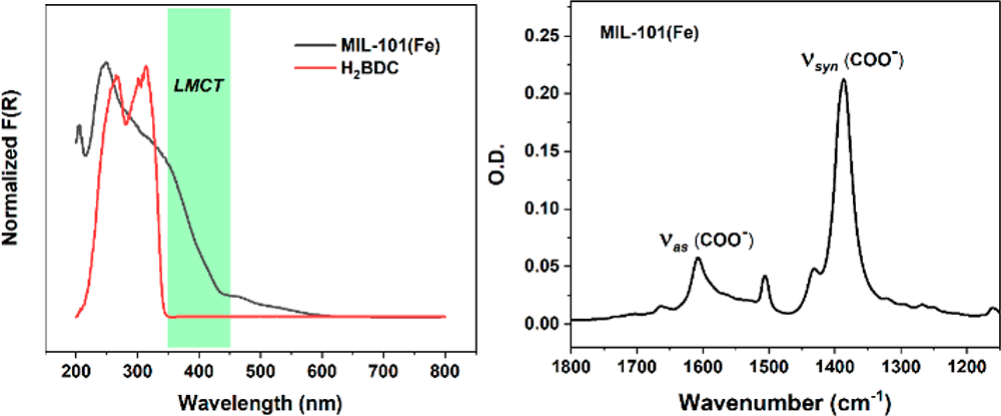
(Left) Diffuse reflectance spectra of MIL-101(Fe) (black) and H_2_bdc (red). (Right) FTIR absorption spectrum of MIL-101(Fe) and characteristic peaks of ν_sym_(COO^–^) and ν_as_(COO^–^) at 1387 and 1606 cm^–1^ are labeled accordingly.

The steady-state IR absorption spectrum (Figure 2, right) of MIL-101(Fe) displays characteristic peaks at 1387 and 1606 cm^–1^, corresponding to the symmetric (ν_sym_) and asymmetric (ν_as_) COO^–^ stretching of the linker in the framework, respectively.^34^ The vibrational modes were also corroborated by the simulated IR spectrum of Fe_3_(μ_3_-O)Cl(H_2_O)_2_(Bz)_6_ as shown in Figure S8 (Bz = benzoate), where the calculated ν_sym_(COO^–^) and ν_as_(COO^–^) modes are at 1439 and 1670 cm^–1^, respectively. Compared to the IR spectrum of H_2_bdc (Figure S9), the most prominent difference is the rise of the ν_sym_(COO^–^) and ν_as_(COO^–^) modes and the decrease of two major peaks centered at 1277 and 1672 cm^–1^, corresponding to the ν(C–O) and ν(C=O) modes of the −COOH group. These changes indicate the formation of the symmetrical bidentate bridging configuration in the MOF where the two coordinated carboxylate oxygens bridge across two Fe atoms from the same Fe_3_O cluster, which is the typical coordination mode of MIL-101(Fe) as shown in the node detail scheme of Figure 1.^35^

In order to probe the LMCT electronic excited states, visible transient absorption (VisTA) spectroscopy was conducted for MIL-101(Fe) with 355 nm excitation.^23^ Shown in Figure 3 are the transient absorption spectra and related kinetic trace measurements at 645 ± 25 nm for MIL-101(Fe). The excited electronic state of MIL-101(Fe) is characterized by a broad excited-state absorption (ESA) from 450 to 700 nm, assigned to an LMCT excited state absorption, as it resembles that of Fe_3_O-Bz in ethanol (Figure S10) and prior reports.^23^ Note that the shown ESA features of MIL-101(Fe) are not the result of scattering, which is corroborated by wavelength-dependent control experiments (Figure S11). Notably, the VisTA spectrum of MIL-101(Fe) is vastly different from that of the linker ester, BDC(OEt)_2_, which displays an excited T_1_* → T_n_* absorption at λ_max_ = 315 nm after 280 nm excitation (Figure S12). Excitation of BDC(OEt)_2_ at longer wavelengths (e.g., 355 nm) resulted in no VisTA spectrum as the π–π* transition does not absorb in this region (in accordance with the DR-UV–vis of the linker ester shown in Figure S13). The visible absorption kinetics of photoexcited MIL-101(Fe) displayed a long-lived biexponential feature, with average lifetimes of 3.25 ± 0.03 and 34.2 ± 0.2 μs (Figure 3). We putatively attribute the short lifetime component to a population of electrons localized in photoreduced Fe^2+^ atoms (λ_max_ ∼ 630 nm) and the long lifetime component to delocalized Fe^2+^ sites throughout the MOF node, congruent with the broad positive absorption at longer time delays.

**Figure 3:**
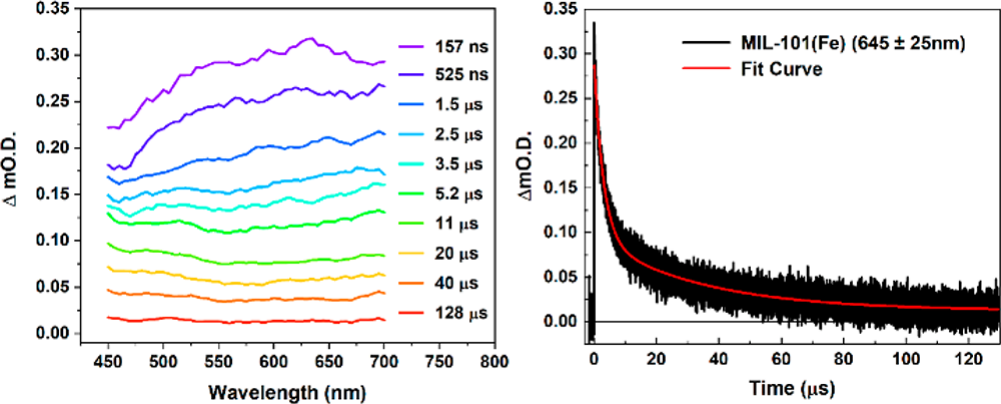
(Left) ns-visible transient absorption spectra of MIL-101(Fe) at varied time delays upon photoexcitation. (Right) selected kinetic trace of MIL-101(Fe) at 645 ± 25 nm (black) and biexponential fit (red). λ_ex_ = 355 nm, 800 μJ cm^–2^.

Time-resolved infrared (TRIR) spectroscopy was employed to explore whether a corresponding dynamic nature of the MOF vibrational modes exists upon electronic photoexcitation. The transient vibrational features of MIL-101(Fe) were probed upon 355 nm pump excitation (Figure 4). Clear ground-state bleaches (GSB) were observed with minima at 1400, 1510, and 1590 cm^–1^, corresponding to the major ground-state absorption peaks, i.e., ν_sym_(COO^–^), ν(C–C), and ν_as_(COO^–^), respectively. ESA peaks were observed at 1190 cm^–1^, 1360 cm^–1^, 1480 and 1710 cm^–1^. The shifted absorptions at 1360 and 1480 cm^–1^ indicate a weakening of the respective bonds [ν_sym_(COO^–^), ν_as_(COO^–^), and ν(C–C)].^36−38^ The relatively small growth centered at 1710 cm^–1^ matches the absorption of carbonyl C=O bond, which was previously reported for MIL-101(Cr) with uncoordinated H_2_bdc acid.^39^ Meanwhile, the ESA centered at 1190 cm^–1^ is the signature of C–O single bond formation.^40^ Importantly, the formation of both C=O and C–O species indicates that the previously symmetrical bidentate bridging configuration between the carboxylate COO^–^ and Fe atoms transitioned to an asymmetrical monodentate mode upon photoexcitation. Notably, the transients gradually recover, meaning that the configurational change (partial deligation event) is reversible (Figure S14). Kinetics traces over the recorded spectral region exhibit monoexponential decays with an average time constant of 28.2 ± 0.4 μs over the probed spectral region (Figure S15). The kinetics of the two most prominent peaks (ESA at 1360 cm^–1^ and GSB at 1400 cm^–1^) are shown in Figure 4 with respective time constants of 21.0 ± 0.5 and 28.8 ± 0.6 μs (ESA traces at 1190 and 1710 cm^–1^ are shown in Figures S16, S17). It is noteworthy that photoinduced decarboxylation^41,42^ was not observed, as transient CO_2_ formation was not detected during the TRIR measurement (i.e., absorption at 2350 cm^–1^ was not observed, Figure S18), which indicates that MIL-101(Fe) could potentially avoid such photoinduced degradation pathways. Additionally, compared to the FTIR spectrum of H_2_bdc, the relatively weaker absorptivity of C=O species probed in photoexcited MIL-101(Fe) strongly indicates the presence of LMCT states (please see SI for a more detailed discussion).

**Figure 4:**
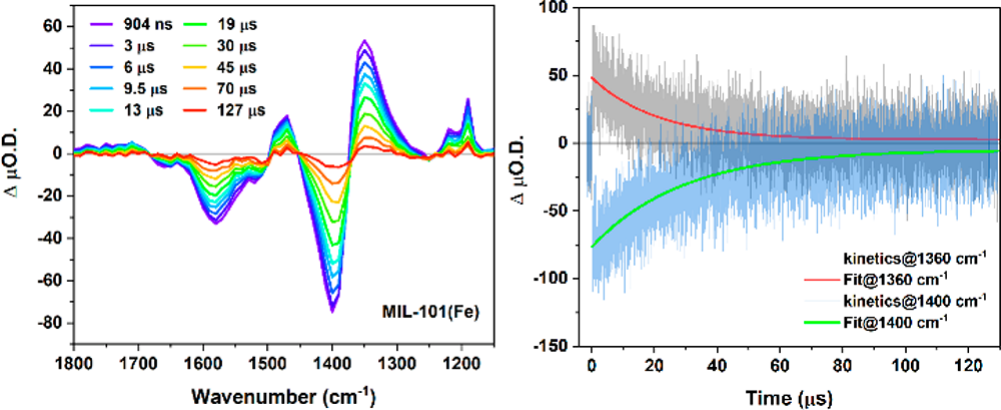
(Left) TRIR difference absorption spectra of MIL-101(Fe) collected at varied time delays after photoexcitation. (Right) kinetics probed at 1360 and 1400 cm^–1^ and corresponding fit curves, with respective lifetimes of 21.0 ± 0.5 and 28.8 ± 0.6 μs. λ_ex_ = 355 nm, 90 μJ cm^–2^.

Moreover, we have carried out VisTA and TRIR for the molecular analogue Fe_3_O-Bz spin-coated on a CaF_2_ substrate as we did for MIL-101(Fe) and similar μs-long features were probed (Figures S19, S20). This suggests that the photoinduced event indeed happens within the coordination structure shared by both systems.

Given the TRIR lifetime is in close agreement with the long lifetime component of VisTA, we associate the photoinduced charge-separated state with the photoinduced transient partial deligation between the carboxylate ligand and Fe_3_O node. Electronic structure calculations support this hypothesis. Analysis of time-dependent density-functional theory (TD-DFT) excitations within a 355 nm window reveals transitions in which electron density in a bonding Fe-carboxylate orbital evolves to an antibonding orbital centered on the Fe^3+^ upon excitation (Figure 5). This electron transfer would initiate deligation of the BDC linker to the node associated with potential bonding transformations as depicted in the scheme of Figure 5.

**Figure 5:**
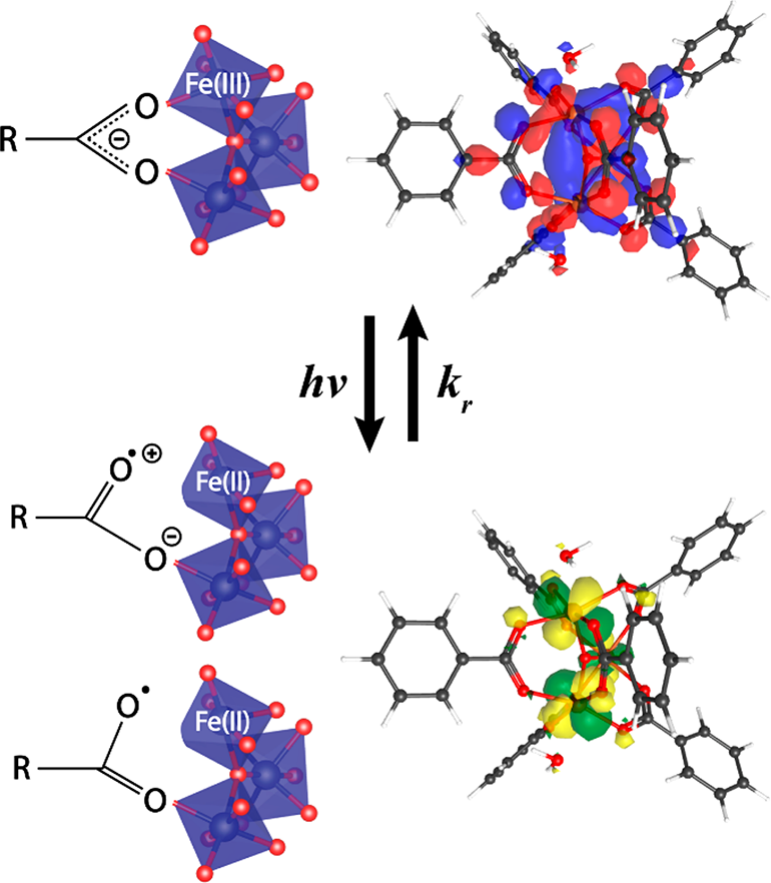
Scheme of the photoinduced phenomenon reported herein in MIL-101(Fe). The molecular orbitals correspond to an electronic transition at 428 nm with a 0.0292 oscillator strength that transfers electron density from a bonding Fe-BDC orbital (top) to a Fe^3+^-centered orbital (bottom).

The presented study represents the first use of both VisTA and TRIR to probe the configurational changes that occur in a carboxylate MOF upon photoinduced electronic transitions. Specifically, the COO^–^-Fe_3_O coordination reversibly switches from a symmetric bidentate bridging configuration to an asymmetric monodentate one with 355 nm excitation and this excited state is sustained for an exceptionally long time (*ca*. 30 μs) before recovering to the initial bidentate ground-state. Complementary VisTA spectroscopy confirmed that the transient bond transformation arises from photoexcitation of an LMCT transition. Electronic-structure calculations identify excitations that afford electronic redistribution, consistent with experimental results. Importantly, the monodentate configuration of the excited state indicates a more pronounced spatial separation between the deligated Fe and O atoms, which prolongs the LMCT state in the system. The reversible ligand dissociation that occurs in MIL-101(Fe) upon photoexcitation grants Fe-based MOFs the capacity to recover their original configurations and states with little-to-no degradation. With its long lifetime, the photoinduced partial deligation of the linker from the Fe_3_O node in MIL-101(Fe) may offer additional catalytically active sites in the MOF matrix, and is likely a notable contributor to the enhancement of observed photocatalytic activity. The correlation between the reported phenomenon and the catalytic properties of MOFs will be an area of future study. Due to the common presence of carboxylate linkers and metal-based nodes in most MOFs, the observations in this work are largely applicable toward the investigations of photocatalytic behaviors occurring in analogous MOF photosystems. In general, the herein-reported reversible photoinduced dynamic phenomenon provides clear structural evidence that bond breakage and physical charge separation leads to ultralong lived excited states in MOFs, thus providing new pathways to promote photocatalytic processes that are free from traditional molecular degradation routes.

## Supporting Information Available

Experimental materials and methods; synthesis procedures; electronic structures calculations; expanded discussion of TRIR data; powder diffraction crystallography; scanning electron microscopy; thermogravimetric analysis; Brunauer–Emmett–Teller analysis; X-ray photoelectron spectroscopy; steady-state and time-resolved spectroscopy (PDF)
