# Supplementary material for: Photoinduced Dynamic Ligation in Metal–Organic Frameworks
Source: J Am Chem Soc. 2023 Dec 27;146(1):101–5. doi: 10.1021/jacs.3c12217 (PMC10785796; doi:10.1021/jacs.3c12217)
Supplement: Supplementary file 1 — ja3c12217_si_001.pdf [file ja3c12217_si_001.pdf]

Supporting Information

Photoinduced Dynamic Ligation in Metal-Organic Frameworks

Qingyu Ye[1], Daniel R. Cairnie[1], Diego Troya[1], Naveen Kumar[1], Xiaozhou Yang[1], and Amanda J. Morris[1]*

1. Department of Chemistry, Virginia Tech, Blacksburg, VA, 24060, USA

Experimental Materials and Methods

Materials. All starting materials and solvents were obtained from commercial sources (Aldrich, Fisher, VWR) and used without further purification, unless otherwise specified.

Powder X-ray Diffraction. A Rigaku Miniflex diffractometer (Cu Kα radiation λ = 1.5418 Å) was used to collect PXRD measurements of MIL-101(Fe) powder to determine crystallinity and phase purity. Samples were analyzed at a 0.05° resolution and a 1.0°/min continuous scanning mode over 2θ = 2–50° on a Rigaku Si510 sample holder disc.

Brunauer–Emmett–Teller (BET) Adsorption Measurements. MIL-101(Fe) (20–50 mg) was loaded into a 12 mm Micromeritics gas adsorption sample cell and activated at 120 °C for 24 h under vacuum. A 3Flex surface analyzer (Micromeritics, Norcross, GA, USA) was used to determine the surface area and pore size of MIL-101(Fe) under N_2.

TGA Measurements. Decomposition temperatures of MIL-101(Fe) (2.306 mg) were determined using a Discovery TGA 5500 (TA Instruments, New Castle, DE, USA), which was held at 100 °C for 1 h to eliminate the solvent before heating over a range of 25–700 °C at 10 °C/min under N_2.

Scanning Electron Microscopy (SEM). SEM images of MIL-101(Fe) particles were collected on a LEO 1550 field-emission scanning electron microscope (Carl Zeiss, Oberkochen, Germany) at 5.0 kV with a 5.0 to 7.7 mm working distance. Samples were prepared by drop-casting the MOF suspended in ethanol onto cut fluorine-doped tin oxide (FTO) slides, which were affixed to SEM stages with double-sided copper tape. The stages were then painted with carbon-conductive adhesive and sputtering-coated with Pt and Pd.

X-ray Photoelectron Spectroscopy (XPS) Measurements. XPS spectra were collected on a PHI 5000 Vera Probe III spectrometer using an aluminum anode X-ray source with photon energy of 1486.6 eV.

Attenuated Total Reflectance-Infrared (ATR-IR) Measurements. ATR-FTIR spectra were obtained using a Varian 670 FT-IR Spectrometer. All spectra were an average of 64 scans for powdered samples and were recorded from 4000 cm^{-1} to 400 cm^{-1} with 2 cm^{-1} resolution.

UV-Vis Solution Absorbance/Diffuse Reflectance Measurements. Absorption measurements were performed with an Agilent Technologies Cary 500 UV-Vis-NIR spectrophotometer equipped with a diffuse reflectance accessory when needed.

Nanosecond TRIR Setup for Film Measurements. Transient infrared absorption measurements were conducted with a Magnitude Instruments inspIRe system. The probe beam was an IR Globar, while the pump source was an in-board diode-pumped solid state Nd:YAG laser outputting the third harmonic wavelength (355 nm) at 6000 Hz. The excited-state kinetics were obtained in 10 cm^{-1} increments from 1150 cm^{-1} to 1800 cm^{-1}, with 6000 laser shots iterated for 50 times at each wavelength and a time window of 131 μs. A total of 2 kinetic sweeps over the range of 1150-1800 cm^{-1} nm were averaged. The outputted pump beam had an area of 1 cm^2 and the excitation energy density was 90 μJcm^{-2}. The sample was rotated periodically throughout the measurements to minimize degradation. Measurements were conducted under a flow of dry air.

Solution-State Nanosecond Transient Absorption Measurements. Time-resolved transient absorption measurements were conducted with an Edinburgh Instruments LP980 laser flash photolysis system. The excitation source was either a frequency-tripled (355 nm) Spectra-Physics Quanta-Ray INDI Nd:YAG laser, operating at 1Hz with a 6-8 ns pulse width, or a dye laser (Sirah CobraStretch) circulating an ethanolic solution of Rhodamine 6G (Exciton, Inc.), tuned to lase at 566 nm. The dye laser emission was passed through a β-barium borate (BBO) crystal to generate a second harmonic (SHG) output of 283 nm and was separated from the fundamental wavelength with a set of Pellin-Broca prisms. The pump source for the dye laser was a frequency-doubled (532 nm) Spectra-Physics Quanta-Ray INDI Nd:YAG laser, operating at 1Hz with a 6-8 ns pulse width. The spectrometer was equipped with an Andor i-Star ICCD camera for steady-state measurements and a Hamamatsu R928 PMT for measuring single wavelength kinetics. The white light source was a pulsed 150 W XBO Xe arc lamp. The average energy per pulse was kept at 1-2 mJ for measurements, unless noted otherwise. Sample stability was monitored via UV-Vis before and after measurements. For spectral absorption mappings, time-zero was defined as the emission signal after the exciting laser pulse disappeared from the emission spectrum. The reported single-wavelength kinetic lifetimes were averaged over multiple trials.

Ultrafast Transient Absorption (ufTA) Measurements. Ultrafast transient absorption measurements were performed with a HeliosFire spectrometer (Ultrafast Systems). The 360 nm pump source was obtained by passing a portion of the 800 nm light generated by a Coherent Astrella ultrafast laser system (1 kHz, 35 fs FWHM) through an optical parametric amplifier (Apollo, Ultrafast Systems). Residual 800 nm light was directed into a mechanical delay stage (EOS, Ultrafast Systems) where the outputted light was focused onto a CaF_2 or sapphire window to generate a white light continuum probe beam. The pump source was chopped to 500 Hz, while the probe beam remained at 1 kHz, so that every other probe pulse had no pump present. The pump beam polarization was perpendicular to the probe beam polarization. The pump beam spot size was determined by placing a pinhole wheel (Thorlabs, PHWM16) at the focus of the beam and a power meter (Thorlabs, PM100D) with a sensor (Thorlabs, S401C) on the other side of the wheel. The diameter of the pinhole was progressively decreased while recording the power. Plotting power versus diameter yielded a quasi-gaussian curves with a $1/e^2$ of ~250 μm. The power hitting the sample was adjusted to an appropriate linear response. The sample cell was placed in a mount

and stirred at 1000 rpm throughout the measurements to minimize sample degradation. The presented spectra are the averages over 3-4 scans with 2 s per point. The data obtained from the ufTA measurements was processed with the SurfaceXplorer software. The worked-up data was plotted with an Origin software package.

Nanosecond Visible Transient Absorption Setup for Film Measurements. For film sample measurements, a Magnitude Instruments enVISion transient absorption system was used. The probe beam was a 150 W Xe arc lamp operated in continuous wave mode, while the pump source was a Magnitude Instruments tunable OPO outputting 355 nm at 50 Hz. The pump beam was focused to a 1 cm^2 area, and the excitation energy density was ~800 μJcm^{-2}. Measurements were conducted under a flow of dry air. The excited-state kinetics were obtained in 5 nm increments from 430-700 nm, with 2000 laser shots at each wavelength and a time window of 128 μs. A total of 2-3 kinetic sweeps from 430-700 nm were averaged. The TA spectra were generated by averaging specific time points in the kinetic traces from 430-700 nm. The displayed spectral time delays (t_d) <1 μs after laser excitation in Figure 3 (main text) was the average of a 50 ns window (e.g. 157 ± 25 ns, 525 ± 25 ns), for 1 μs < t_d < 10 μs the average time window was 200 ns, and for t_d > 10 μs the average time window was 1 μs. The TA spectra were all subjected to a 3-point smooth (moving average) across the wavelengths. The kinetic trace in Figure 3 (main text) was obtained by averaging the kinetics across a 50 nm window (620-670 nm, 645 ± 25 nm) and clarified with a 3-point smooth (moving average). The spectra and kinetics were processed with MATLAB and the kinetics were fit with a biexponential model in Origin data analysis software.

Electronic Structure Calculations

Time-dependent density-functional theory (TD-DFT) calculations were carried out to further understand the photophysics of MIL-101(Fe). From the crystal structure, a cluster model was produced consisting of a central Fe_3O moiety capped by 6 benzoate ligands. The coordination shell of the Fe(III) atoms is saturated by 2 aqua and 1 chloride ligand (Figure S21). Geometry optimization and vibrational frequency calculations on this $Fe_3(O)(OOCBz)_6(H_2O)_2Cl$ model were performed with the B3LYP method and 6-31G* basis set as implemented in the Gaussian09 suite. All even spin multiplicities up to 16 were probed in the calculations, and the spin state in which all 15 unpaired electrons are parallel was of lowest energy. The calculated IR spectrum of this

ground state is shown in Figure S8. Using the optimum geometry with hexadectet spin multiplicity, TD-DFT calculations at the same level of theory were performed to identify excited states within the 355 nm wavelength excitation window of the experiment (calculations with an antiferromagnetic wavefunction of sextet spin mutiplicity provided comparable results). 102 excitations were found in the excitation window. The character of the excitations with largest oscillator strengths were analyzed. The excitation with the largest oscillator strength (f = 0.0292, at 427.56 nm) is formed in part by an electronic transition between a bonding orbital that coordinates a benzoate unit to a Fe(III) atom and an antibonding orbital centered on the Fe(III) atom (Figure 5 in main text). An electronic transition between those two orbitals would act to unclip the benzoate from a Fe atom upon excitation.

MIL-101(Fe) Synthesis.

In a typical synthesis of MIL-101(Fe) following the reported procedure (1), 2.45 mmol iron chloride hexahydrate ($FeCl_3•6H_2O$) and 1.24 mmol benzene-1,4-dicarboxylic acid (H_2bdc) were added to 15 mL *N,N*-Dimethylformamide (DMF) and the mixture was heated at 110 °C for 20 h in a Teflon-lined stainless steel Parr bomb. The synthesized brown solid was recovered by centrifugation, washed with ethanol (60 °C for 3 h, 2 times) and then dried in a vacuum oven at 60 °C for 12 hours.

Fe_3O-Bz Cluster Synthesis. The model Fe_3O-Bz cluster ($[Fe_3O(OOCBz)_6(H_2O)_3]ClO_4$) was synthesized according to a previous procedure.[1] An $FeCl_3•6H_2O$ solution (13.5 g, 0.05 mol) in water (50mL) was added slowly to an aqueous solution (300 mL) of $Ph(CO)_2Na$ (25 g, 0.17 mol). A pink-tan precipitate of Fe-(OOCBz) was formed, filtered, washed with water and dried in a 50 °C vacuum oven overnight. A 2.0 g portion of the recovered solid was then dispersed in 40 mL of anhydrous ethanol. To the dispersion, a 20% $HClO_4$ aqueous solution (6.0 g, 0.06 mol) was slowly added, and the suspension dissolved. The resulting solution was heated on a steam bath for ~10 min and filtered hot. Boiling water (30 mL) was added to the filtrate and an orange solid precipitated. The precipitate/solution was cooled in a freezer for 2 h to maximize precipitation. The precipitate was gravity filtered and washed with small aliquots of a 1:1 (v/v) water-acetone solution (~15 mL total). The filtered orange solid was placed in a 50 °C vacuum oven overnight and then used as-is for measurements.

MIL-101(Fe) Spin-coating procedure for transient absorption measurements. A MIL-101(Fe) suspension was prepared by dispersing the synthesized particles in ethanol (6% w/w). Prior to spin coating, the CaF_2 substrates (diameter = 25.4 mm, 1 mm thick, Crystran Ltd.) were cleaned with O_2 plasma for 15 min. The MIL-101(Fe) film was prepared by spin-coating the alcoholic suspensions of MIL-101(Fe) onto the substrate at 1500 rpm to 4500 rpm for 30 s. The volume of the suspension used in each spin coating was 100 μL. The spin-coating process was repeated for 1 time to obtain thicker films. The film was annealed at 200 °C for 10 min after each deposition.

Discussion: TRIR evidence suggesting LMCT

The more detailed spectroscopic analysis indicating the presence of LMCT is discussed here. IR spectroscopy has unique sensitivities not only to the bond type/strength but also the charge magnitude of the probed dipole. More specifically, the absorptivity ε of a probed dipole $\vec{\mu}$ is positively corelated to the charge magnitude q:

$$\vec{\mu} = q \cdot \vec{r}$$

$$\varepsilon = \left(\frac{d\vec{\mu}}{d\vec{r}}\right)^2$$

Where $\vec{r}$ is the displacement vector representing the spatial separation of the charge q (the direction is from the negative charge to the positive charge).

In our case, if the LMCT does happen upon photoexcitation, we should observe a change in absorptivity of certain dipole species (chemical bonds) at this charge-separated state, since the change in local electron density could be reflected by the change in the value of q. In fact, by comparing the relative absorption intensity of C=O/C-O in the FTIR spectrum of H_2bdc and the TRIR spectrum of MIL-101(Fe), it is evident that the C=O absorptivity is much smaller in photoexcited MIL-101(Fe) than in ground-state H_2bdc. This drastic drop in absorptivity is a strong

indicator of electron transfer from carboxylate to Fe-oxo node in our sample, which further validates the presence of LMCT state.

Supporting Experimental Data

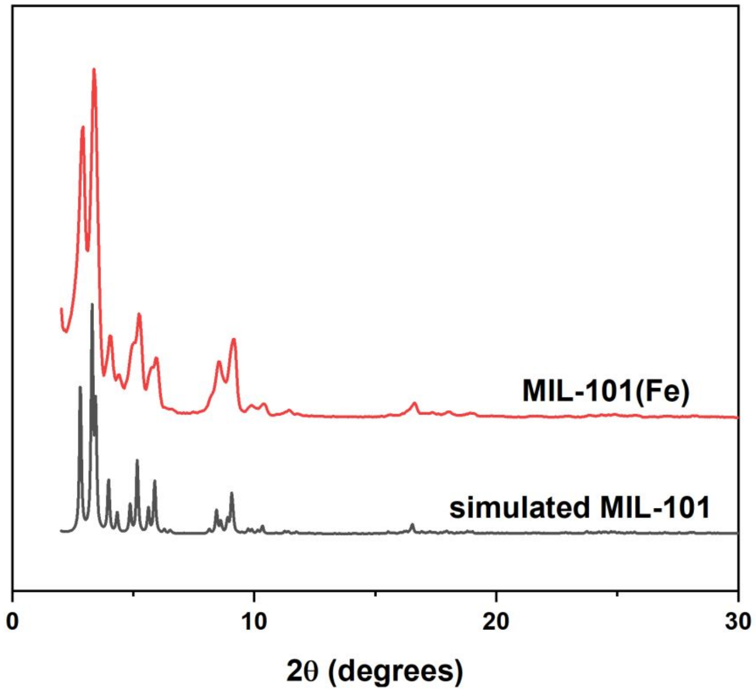

Figure S 1. PXRD of as-synthesized MIL-101(Fe) and simulated pattern of MIL-101 structure.

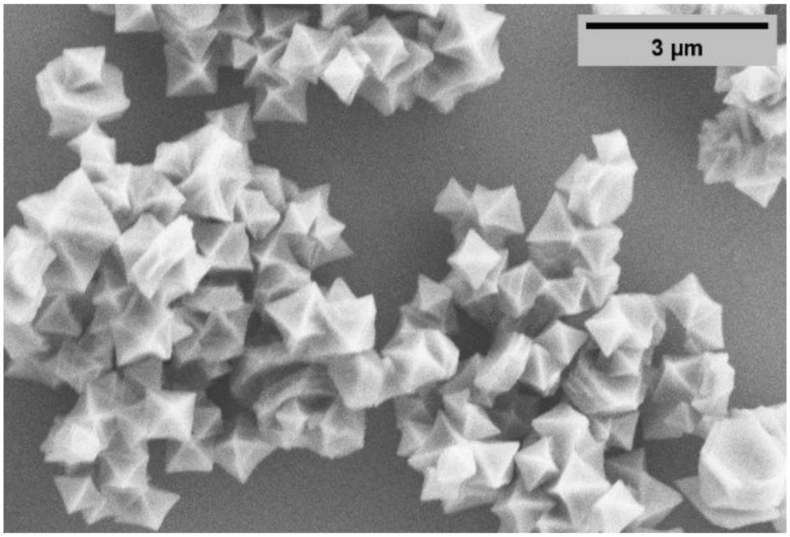

Figure S 2. SEM image of as synthesized MIL-101(Fe) microcrystals.

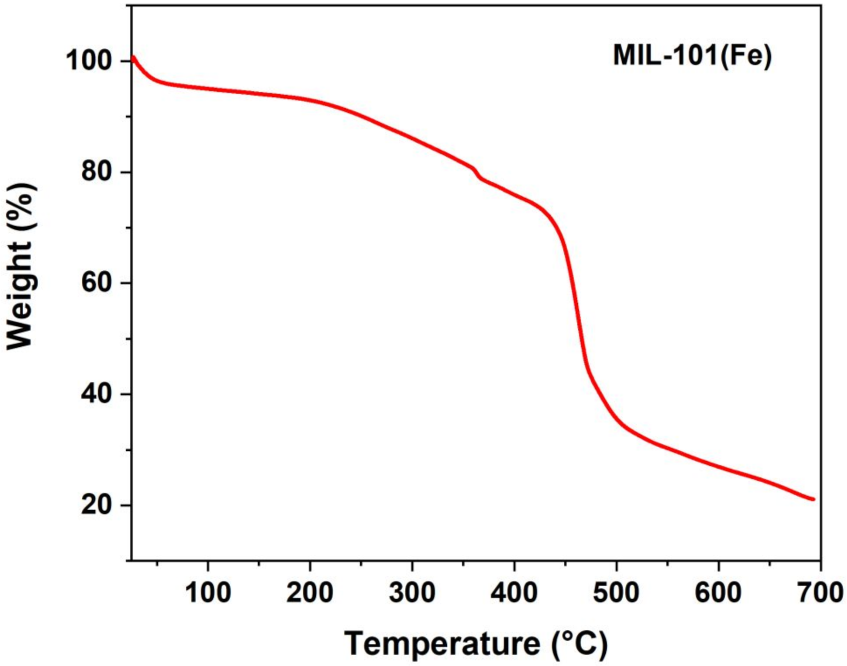

Figure S 3. Thermogravimetric analysis (TGA) of the as-synthesized MIL-101(Fe), showing a decomposition point of ca. 450 °C.

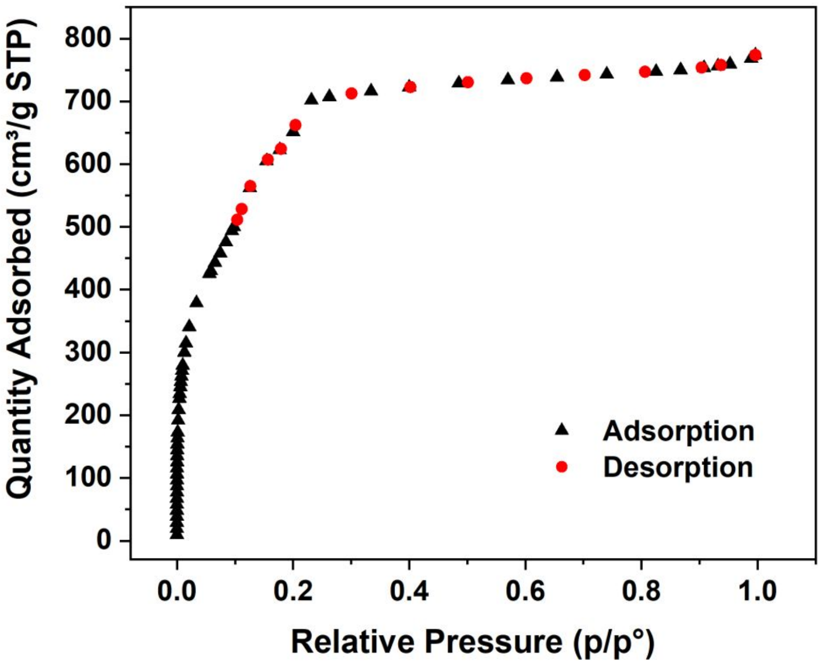

Figure S 4. N_2 adsorption and desorption isotherm of the prepared MIL-101(Fe). The Brunauer–Emmett–Teller (BET) surface area is calculated to be 2234 m^2/g.

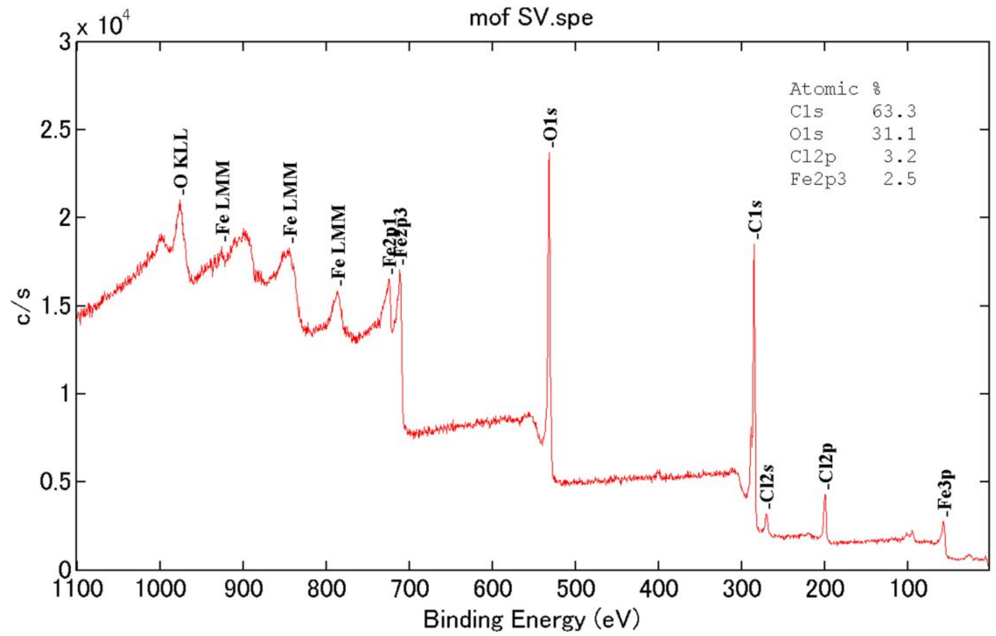

Figure S 5. Full XPS survey spectrum of MIL-101(Fe).

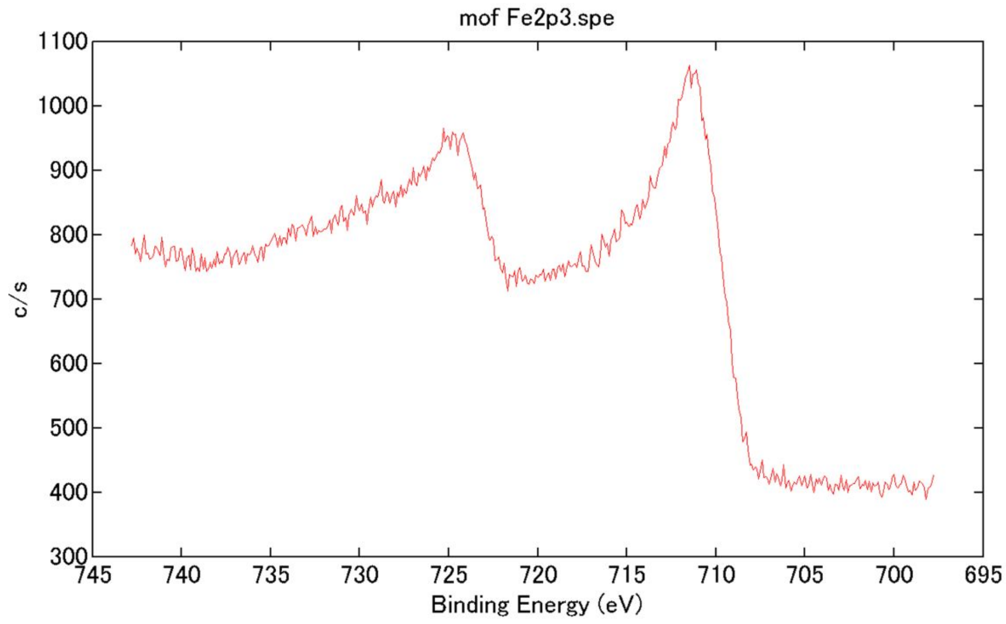

Figure S 6. High-resolution XPS spectrum of the Fe-2p3 orbital in MIL-101(Fe).

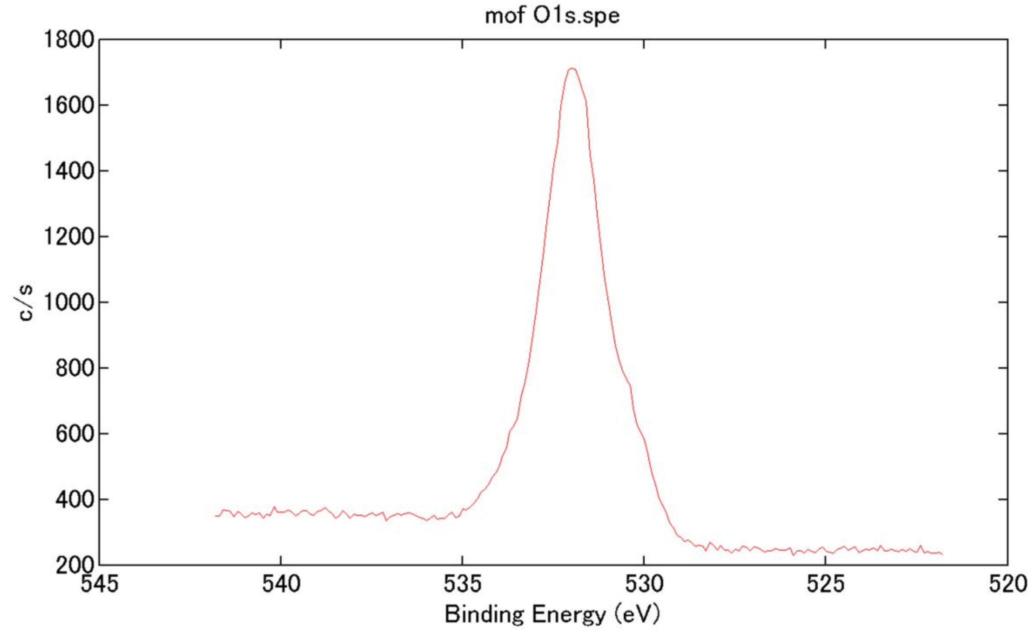

Figure S 7. High-resolution XPS spectrum of the O-1s orbital in MIL-101(Fe).

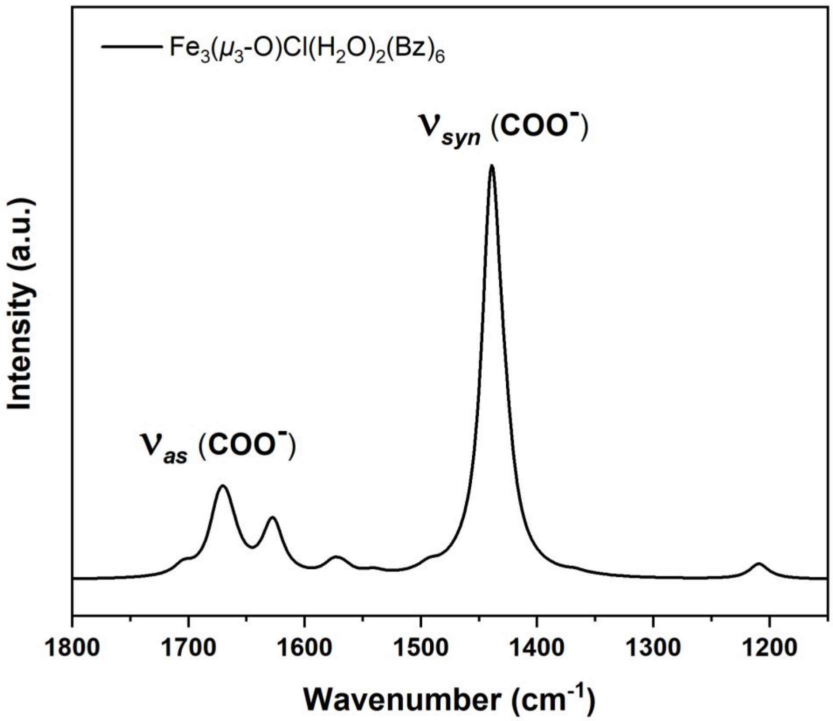

Figure S 8. Simulated IR transmission spectrum of $Fe_3(\mu_3\text{-}O)Cl(H_2O)_2(Bz)_6$ (Note: Bz = benzoate).The $\nu_{syn}(COO^-)$ and $\nu_{as}(COO^-)$ modes are calculated to be centered at 1439 cm^{-1} and 1670 cm^{-1}, respectively.

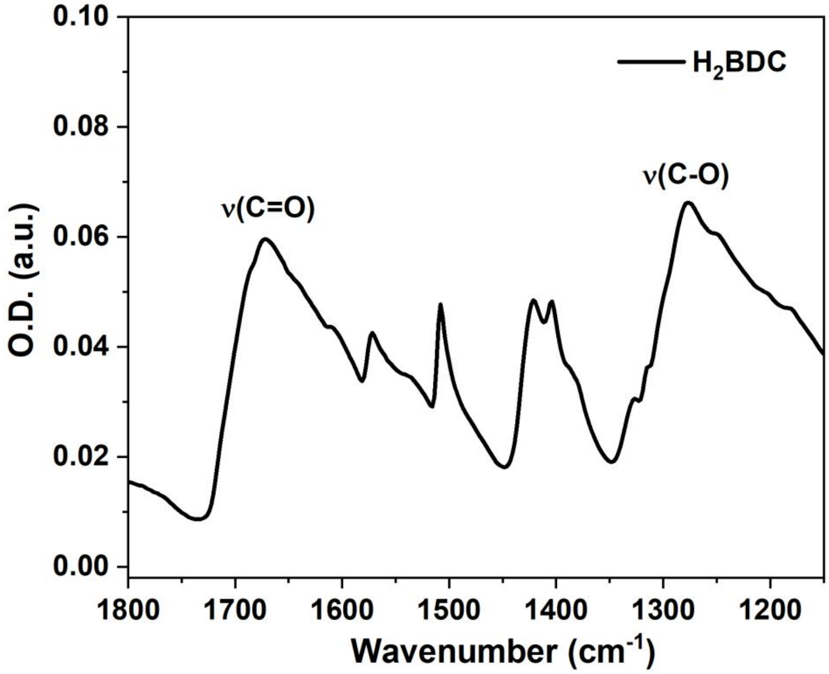

Figure S 9. ATR-FTIR absorption spectrum of H_2bdc. Note the two major peaks centered at 1277 cm^{-1} and 1672 cm^{-1}, corresponding to the ν(C-O) and ν(C=O) modes of -COOH group, respectively.

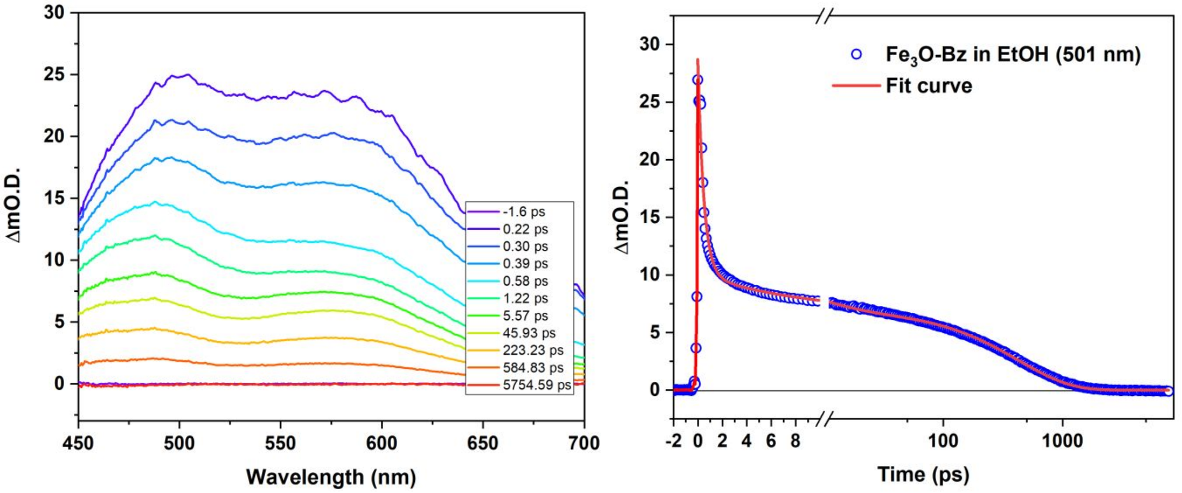

Figure S 10. ***(Left)*** *ultrafast TA spectrum of Fe_3O-Bz over time (purple to crimson) in ethanol. The excited state absorption spectra associated with an LMCT transition in Fe_3O-Bz is characterized by a broad full-spectrum absorption feature.* ***(Right)*** *ultrafast TA kinetic decay curve of Fe_3O-Bz (blue circles) at 501 nm, with the kinetic fit shown in red. The decay lifetimes obtained from the fit were 489 ± 16 fs, 8.4 ± 1.0 ps, 428 ± 15 ps. The first two lifetimes are likely associated with internal conversion from a higher-energy LMCT state to the lowest excited LMCT state, and vibrational cooling of the hot LMCT state, while the third lifetime is due to recombination of excited state carriers with the ground state. λ_{ex} = 400 nm, 7.3 μJ/pulse.*

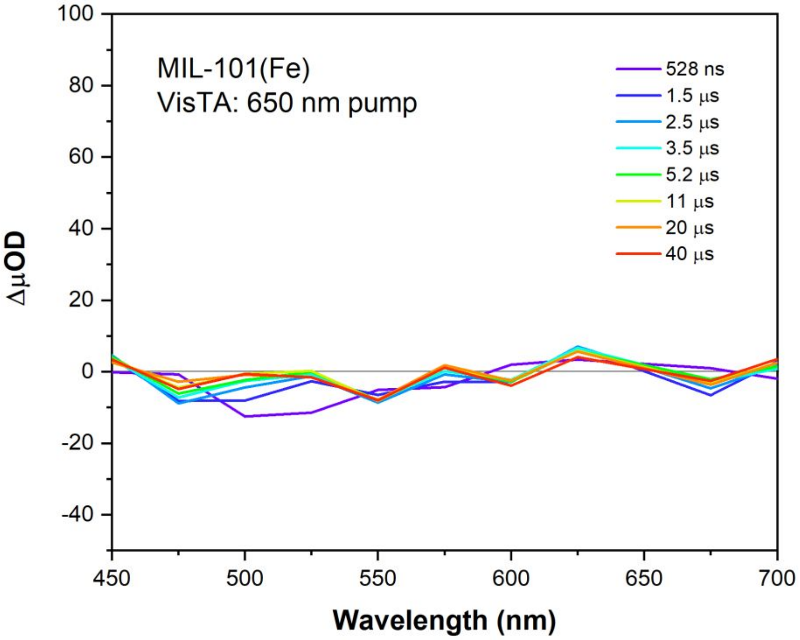

Figure S 11. Vis-TA measurement of MIL-101(Fe) film with 650 nm laser pump. Since the MOF is not electronically excited with 650 nm photons, no significant transient absorption signals are recorded, in stark contrast to the same sample upon 355 nm excitation. This confirms that the transient features of MIL-101(Fe) under 355 nm excitation indeed correspond to the absorptions of the electronically excited states in the system instead of scattering. λ_{ex} = 650 nm, 80 μJ/pulse.

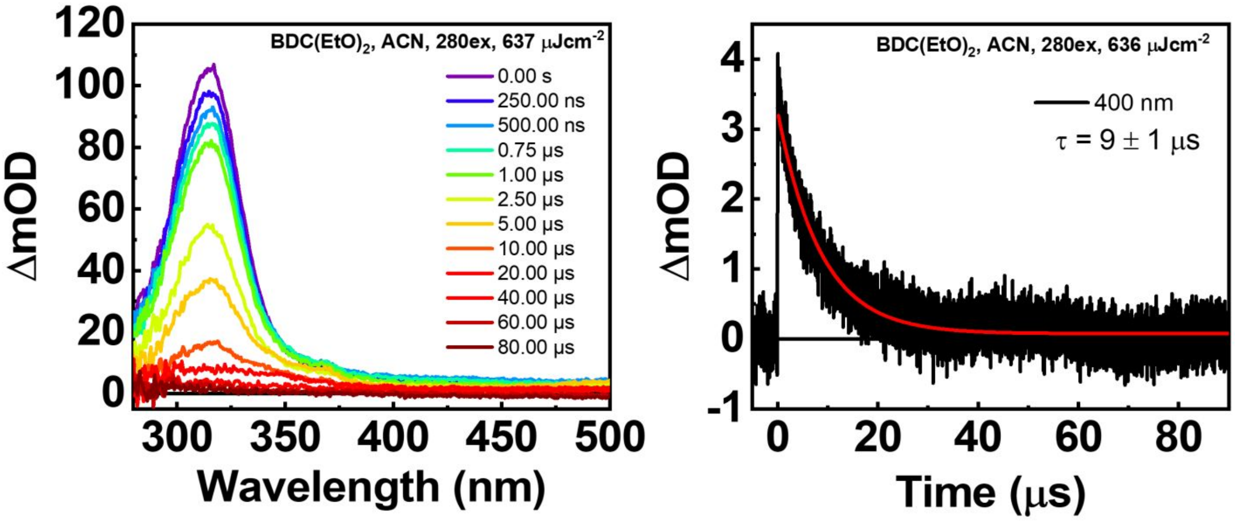

Figure S 12. ***(Left)*** *nsTA spectrum of bdc(OEt)$_2$ over time (purple to crimson) in ACN. The triplet absorption spectrum of is characterized by a strong peak at 315 nm followed by a broad tail out to 500 nm.* ***(Right)*** *triplet decay trace of BDC(OEt)$_2$ from nsTA measurements at 400 nm, with the kinetic fit shown in red. τ = 9 ± 1 μs. λ_{ex} = 280 nm, 637 μJcm^{-2}.*

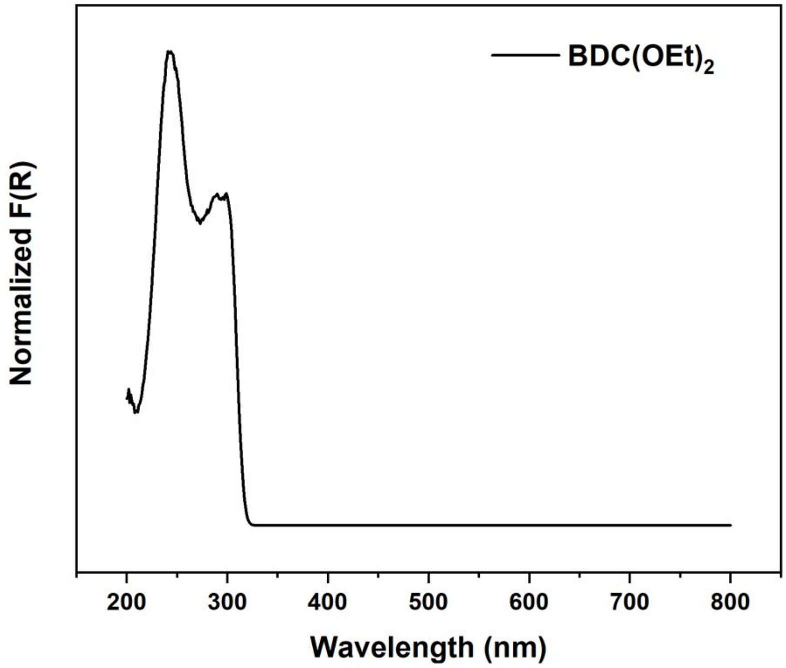

Figure S 13. DR-UV-Vis spectrum of bdc(OEt)$_2$.

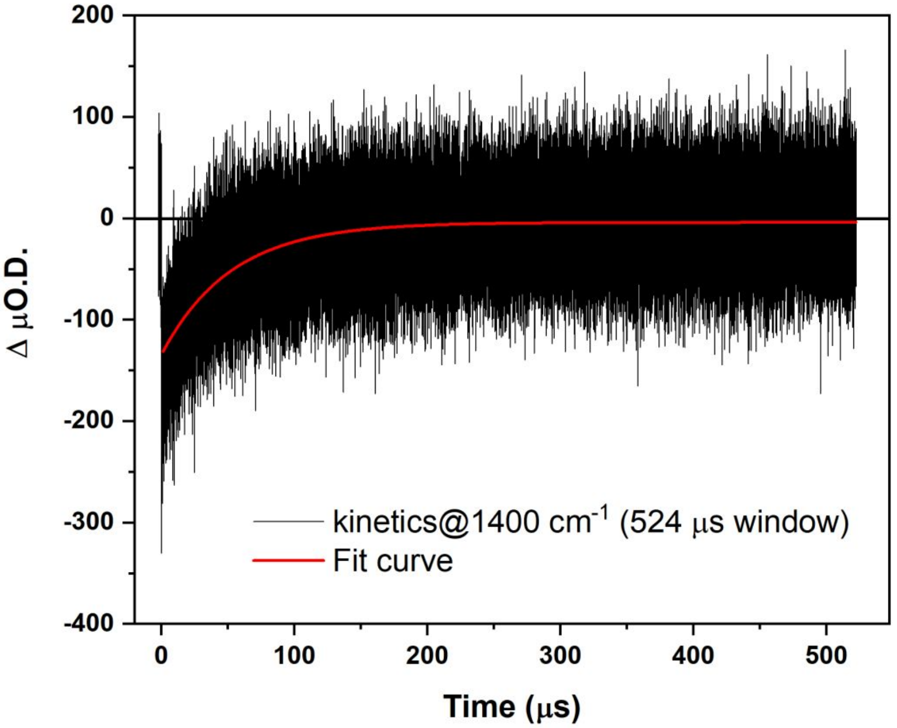

Figure S 14. Kinetic trace probed at 1400 cm^{-1} for MIL-101(Fe) with 524 μs window (with fit time constant of 52.2 ± 0.7 μs, λ_{ex} = 355 nm, 90 μJcm^{-2}). Notice how the bleaching signal gradually recovers to zero.

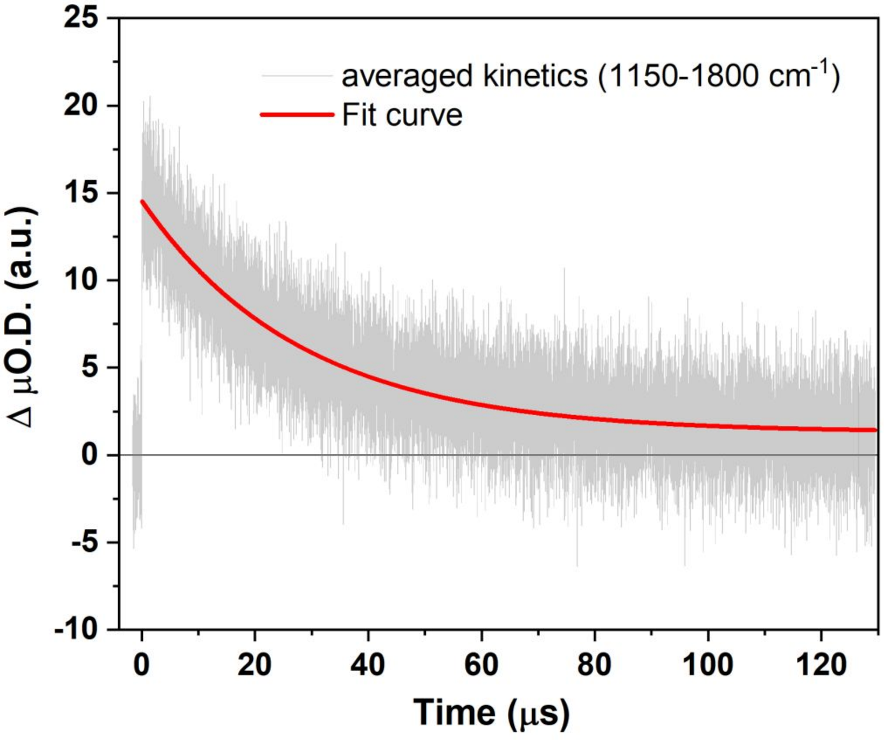

Figure S 15. The TRIR kinetics spectrally averaged over the probed range (1150-1800 cm^{-1}; kinetic values of negative signal regions shown in Figure 4 were reversed before the spectral average), with fit time constant of 28.2 ± 0.4 μs. λ_{ex} = 355 nm, 90 μJcm^{-2}.

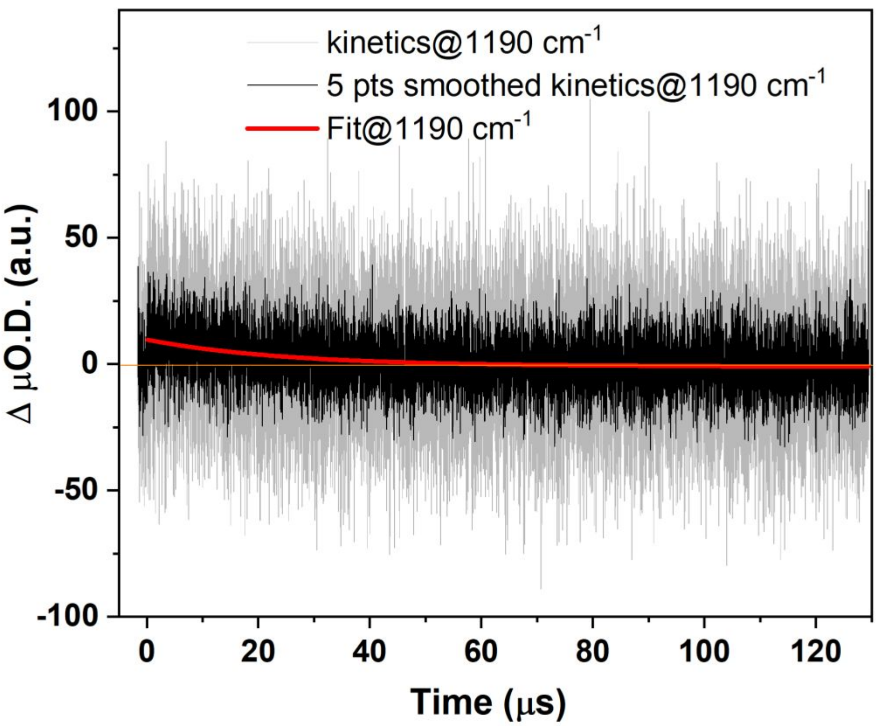

Figure S 16. The ESA kinetic trace (with 5 points adjacent-average smoothed kinetic curve shown in black for clearer illustration) corresponding to C-O formation at 1190 cm^{-1}, with fit time constant of 24.9 ± 4.3 μs. λ_{ex} = 355 nm, 90 μJcm^{-2}.

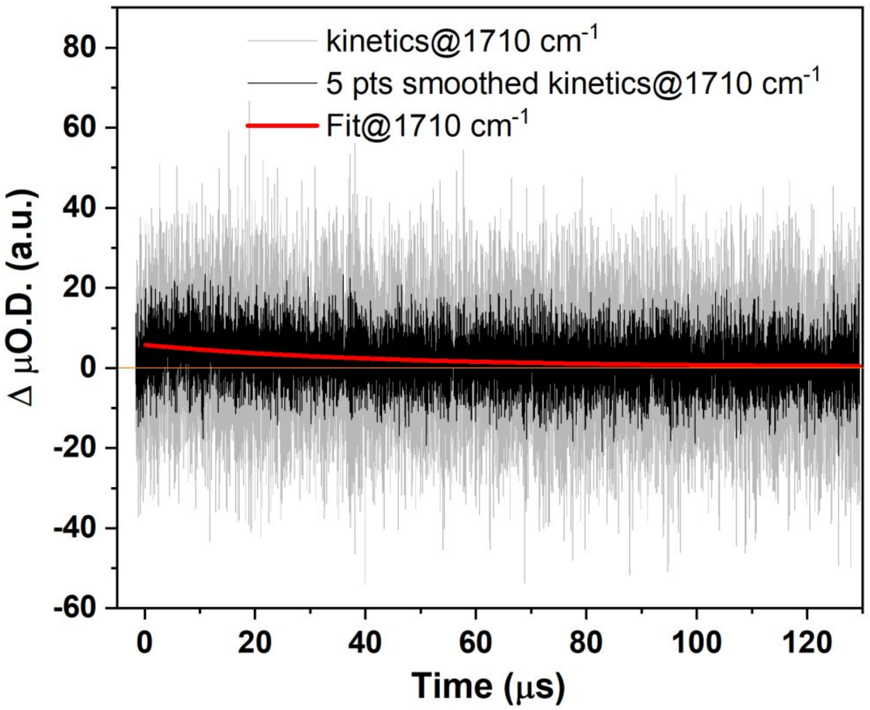

Figure S 17. The ESA kinetic trace (with 5 points adjacent-average smoothed kinetic curve shown in black for clearer illustration) corresponding to C=O formation at 1710 cm^{-1}, with fit time constant of 42.3 ± 10.4 μs. λ_{ex} = 355 nm, 90 μJcm^{-2}.

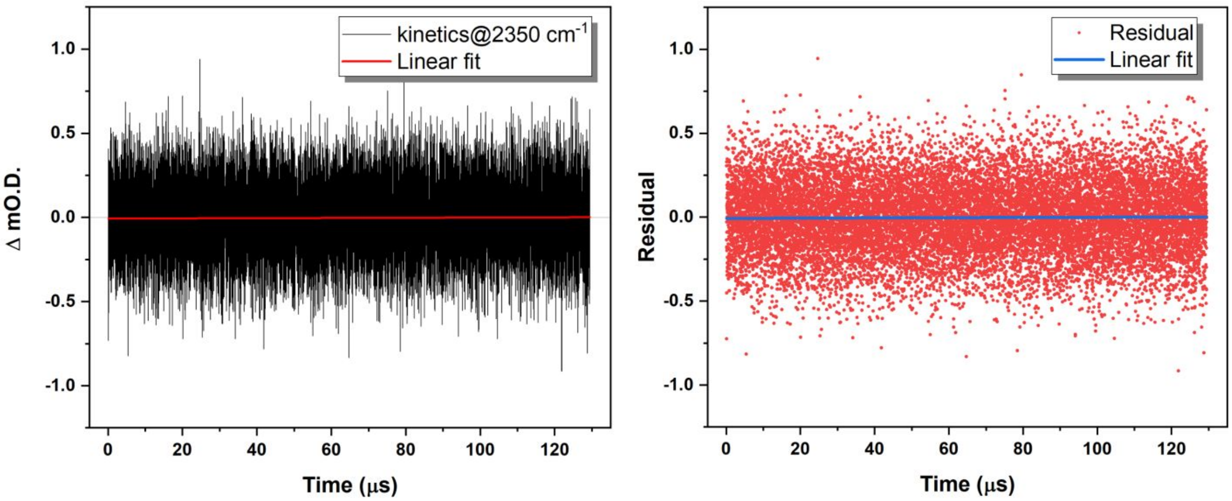

Figure S 18. TRIR kinetics of MIL-101(Fe) at 2350 cm^{-1} on the left (with fit residue plot on the right side), where CO_2 is known to absorb strongly. The lack of signal in this region indicates that CO_2 is not being formed in situ. λ_{ex} = 355 nm, 90 μJcm^{-2}.

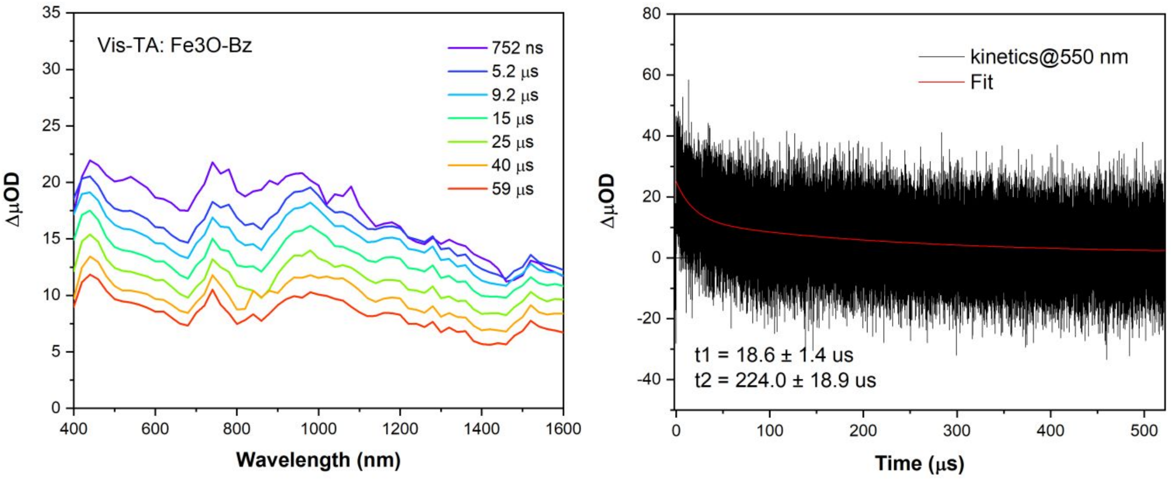

Figure S 19. Vis-TA measurement on Fe_3O-Bz cluster film on CaF_2 window. (Left) difference absorption spectra of Fe_3O-Bz film in the Vis-NIR range collected at varied time delays after 355 nm photoexcitation. (Right) kinetics probed at 550 nm and corresponding fit curve, with respective lifetimes of 18.6 ± 1.4 μs and 224.0 ± 18.9 μs. λ_{ex} = 355 nm, 90 μJcm^{-2}. The different lifetime components could potentially correspond to the distinct species presented in the cluster system as the result of geometrical distortion.[2]

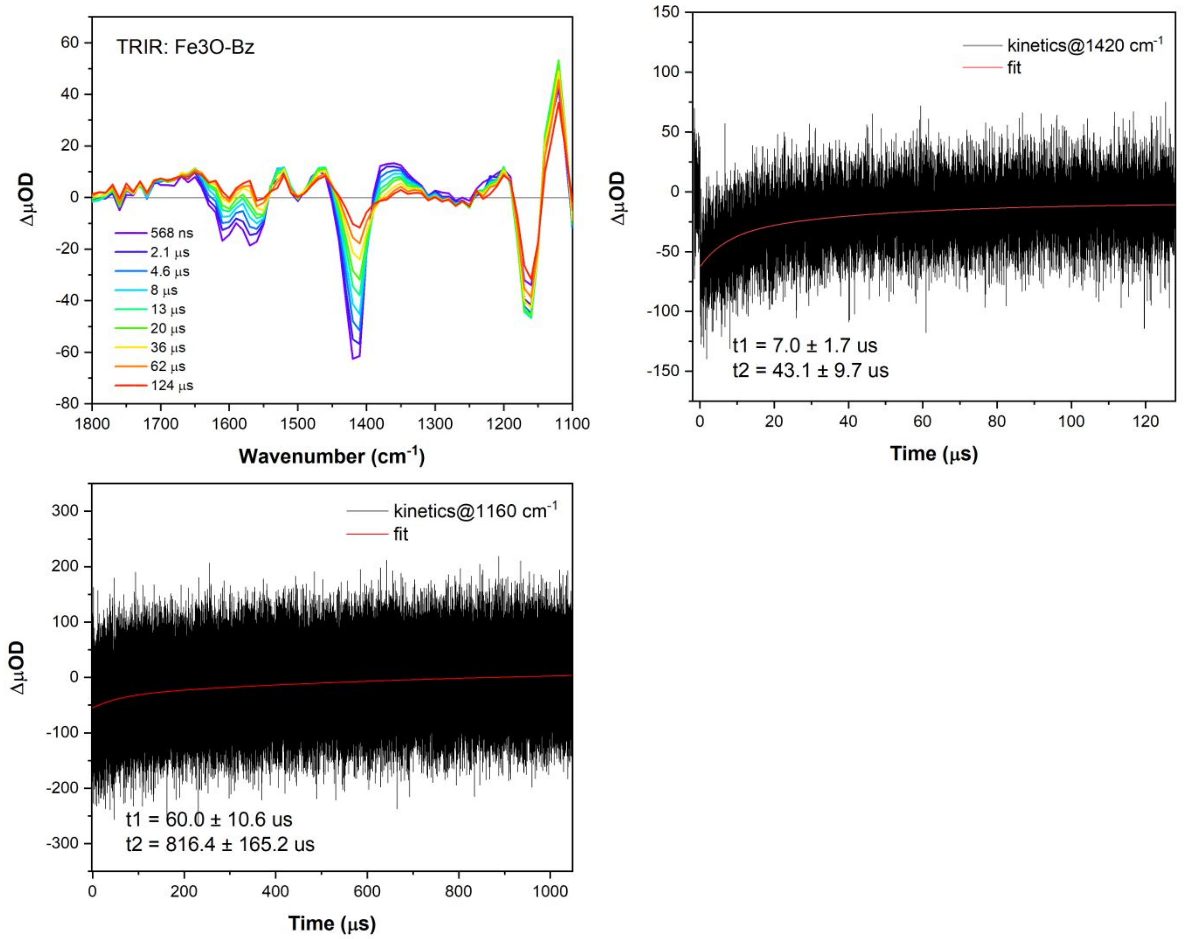

Figure S 20. TRIR measurement on Fe_3O-Bz cluster film on CaF_2 window. (Upper left) difference absorption spectra of Fe_3O-Bz film collected at varied time delays after 355 nm photoexcitation. (Upper right) kinetics probed at 1420 cm^{-1} and corresponding fit curve, with respective lifetimes of 7.0 ± 1.7 μs and 43.1 ± 9.7 μs. (Lower left) kinetics probed at 1160 cm^{-1} and corresponding fit curve, with respective lifetimes of 60.0 ± 10.6 μs and 816.4 ± 165.2 μs. λ_{ex} = 355 nm, 90 μJcm^{-2}. The different lifetime components could potentially correspond to the distinct species presented in the cluster system as the result of geometrical distortion.[2]

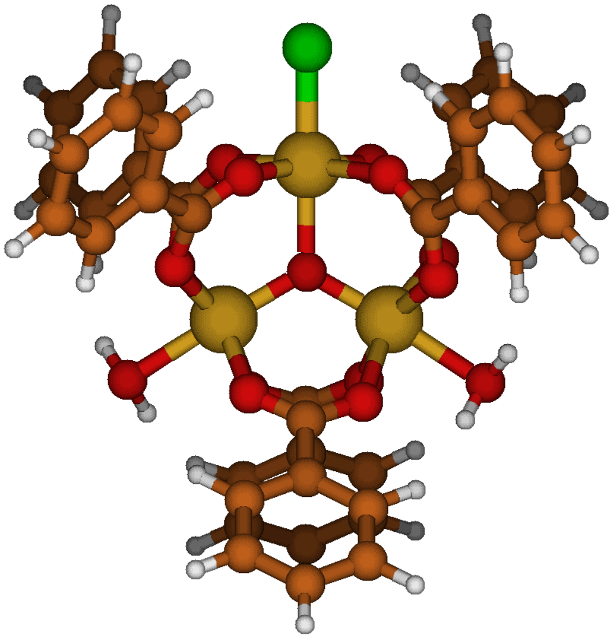

Figure S 21. Optimum geometry of the $Fe_3(O)(OOCBz)_6(H_2O)_2Cl$ model used in the electronic structure calculations (B3LYP/6-31G, hexadectet spin multiplicity). Fe: gold, O: red, H: white, Cl: green, C: brown.*

References

1) Gecgel, C.; Simsek, U. B.; Gozmen, B.; Turabik, M. J. Iran. Chem. Soc. 2019, 16 (8), 1735–1748.
2) Sowrey, F. E. et al. Spin frustration and concealed asymmetry: structure and magnetic spectrum of [Fe3O(O2CPh)6(py)3]ClO4·py. J. Chem. Soc. Dalton Trans. 862–866 (2001).
